# Proactive process evaluation of precision medicine platforms: a roadmap

**DOI:** 10.1136/bmjhci-2025-101434

**Published:** 2025-07-20

**Authors:** Kathrin Cresswell

**Affiliations:** 1 Usher Institute, The University of Edinburgh, College of Medicine and Veterinary Medicine, Edinburgh, UK

**Keywords:** Artificial intelligence, Data Science, Health Information Systems, Health Services Research

## Abstract

**Background:**

Precision and genomic medicine have significant potential to improve population health. However, despite rapid technological development and increasing data complexity, practical applications of precision medicine remain limited. There is also a lack of evaluation of unintended consequences and a failure to use theory-based implementation frameworks to manage risks and ensure sustainability.

**Methods:**

This work provides a conceptual overview of evaluation challenges related to precision medicine platforms, based on existing literature. It proposes a theory-informed proactive process evaluation framework to guide the development and assessment of these platforms.

**Results:**

The proposed framework considers infrastructural, socio-organisational and system-level factors. It raises key questions, such as: How will platforms integrate with existing infrastructures? How will they transform care pathways and the delivery of care across settings?

**Conclusions:**

Rapid technological advances challenge markets and regulatory environments. Agile evaluation approaches are crucial for building a sustainable innovation ecosystem for precision medicine platforms.

## Introduction

 Precision medicine (PM) has significant potential to contribute to the development of a truly learning health system where data-driven approaches guide care provision and treatment decisions.^1^ PM encompasses various inter-related concepts, including the identification and targeting of the causal mechanisms underlying diseases (which may include genomic medicine to understand aetiology), and the implementation of strategies tailored to address root causes of disease informing clinical or public health decisions customised to individual circumstances (box 1).^2^ PM approaches and techniques are increasingly integrated into PM platforms, often incorporating multiple interventions or technologies, such as genomics and artificial intelligence (AI) (eg, image analysis, tissue sample analyses).^3^,^7^

Box 1 **Definitions of terms**

Artificial intelligence (AI)—‘The capacity of computers or other machines to exhibit or simulate intelligent behaviour; the field of study concerned with this. In later use also: software used to perform tasks or produce output previously thought to require human intelligence, especially by using machine learning to extrapolate from large collections of data.’^27^

Precision medicine—‘… the tailoring of medical treatment to the individual characteristics of each patient. It does not literally mean the creation of drugs or medical devices that are unique to a patient, but rather the ability to classify individuals into subpopulations that differ in their susceptibility to a particular disease, in the biology and/or prognosis of those diseases they may develop, or in their response to a specific treatment (page 125).’^47^

Precision medicine platform—A platform comprising inclusion and analysis of various data sources forming the basis for precision medicine approaches.^48^

Precision prevention—Public health strategies designed to tailor interventions according to specific population characteristics and risk factors.^49^

Genomic medicine—‘…an emerging medical discipline that involves using genomic information about an individual as part of their clinical care (eg, for diagnostic or therapeutic decision-making) and the health outcomes and policy implications of that clinical use. Already, genomic medicine is making an impact in the fields of oncology, pharmacology, rare and undiagnosed diseases, and infectious disease.’^50^

Implementation science—Social science-based approaches seeking to understand how new interventions are implemented and adopted.^51^

Formative process evaluation—A specific method under the implementation science umbrella. Social science-based approaches to studying the way interventions are developed, implemented and adopted. This information is then used to inform intervention optimisation to ensure maximum chances of sustained adoption.^52^

However, despite clear potential, there is still a dearth of evidence regarding the actual impact of PM on the safety, quality and efficiency of care.^8^ PM platforms consisting of multiple technological components are in nascent stages and currently primarily used for research purposes (eg, to help gather patient information for trials). They have yet to be integrated into health and care delivery processes. Initiatives also often focus on PM platform development in single institutions,^2^ with adoption, optimisation, scalability and sustainability considerations frequently being an afterthought. This is despite a significant empirical evidence base indicating that social, organisational and health system processes can make or break technology implementation. There are also significant risks associated with unintended consequences of implementing new health information technology (HIT).^9^,^11^ There are therefore missed opportunities to detect and address potentially adverse consequences, increasing the risk that PM platforms are not effectively adopted or fail to scale.

The translation of complex interventions into practice is known to be difficult.^12^ This poses a challenge for fast-developing technological interventions such as PM platforms. Efforts to effectively integrate agile process evaluation methods into PM platform developments are therefore crucial but remain insufficiently applied in practice.^12^ This work provides a starting point for how such evaluations may be conceptualised.

## Evaluation challenges associated with PM platforms

Previous work has explored the evaluation challenges associated with precision prevention interventions, and many of these also apply to PM platform evaluations (box 2).^13^

Box 2 **Challenges assessing impacts and processes surrounding precision medicine interventions**

**Challenges measuring the impact of precision medicine interventions**

- Relevance and fit of external data sources.
- Randomised controlled trials may not be ethical.
- At what level to measure impact (individual, community, societal or a combination).
- Cost-benefit analysis (indirect benefits, cost of data processing).

**Challenges assessing processes surrounding precision medicine interventions**

- Integration with clinical decision-making.
- Evolving nature of interventions, scientific knowledge, needs and environments.
- Contextual variations and use cases (what works in one context may not necessarily work in another).
- Ethical considerations.

Evaluation of interventions including genomic testing poses additional challenges. For instance, when assessing outcomes in the context of preventive activities, it becomes inherently difficult to measure the efficacy of such interventions, and the small populations typically involved in genomic testing can limit generalisability of findings.^14^ It is also difficult to assess: the downstream value of PM interventions, incidental findings where unexpected genetic variations may have potential health implications, and the broader value or risks of tests, including their impact on family members.^12 15^

## Developing theory-informed proactive process evaluation methods of PM platforms

The application of theory-informed HIT evaluation frameworks in PM and genomic medicine is limited, with many existing evaluation studies relying on quantitative methods.^16^ There is also a bias towards exploring micro-factors including organisational-level analysis,^17^ while neglecting broader technological infrastructure and health system considerations.^18^ A recent review of genomic medicine studies further found limited leveraging of existing models, and a wide variation in outcome measures, hindering the extraction of meaningful evidence across studies.^19^

The Implementing GeNomics In pracTicE (IGNITE) network, a collaboration aiming to facilitate the implementation of genomic medicine in practice, is a notable exception. The group has developed a specific framework tailored to evaluate genomic medicine interventions, which takes these factors into account and presents an important step towards theory-informed process evaluation.^20^ These efforts now need to be built on.

**Figure 1:**
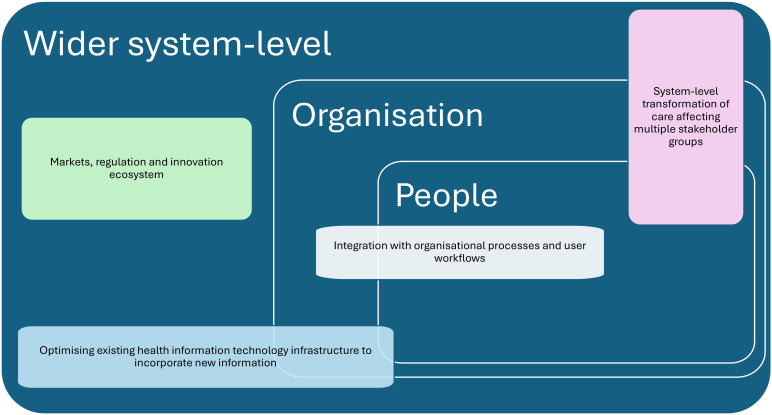
System-level, organisational and people components that need to be considered when evaluating precision medicine platforms.

The model below builds on the important work of the IGNITE network (figure 1). It includes infrastructural, socio-organisational and system-level dimensions that process evaluations of precision and genomic medicine interventions need to consider. The following paragraphs will explore individual components of the model, drawing on PM platforms as a use case, as this is likely to be the most complex form of intervention in this area.

### Optimising existing HIT infrastructures to incorporate new information

Planning how a new PM platform will integrate with existing information infrastructures is often neglected in the development stages, but a crucial aspect of implementation and adoption of such technologies in real-world settings. Current electronic health records (EHRs) do not include provisions for incorporating genomic information.^2^ For information to be integrated into routine care provision, there is therefore a need for EHR systems to either incorporate genomic data directly or to extract relevant information from genomic sources.^21^ This will require close collaboration with existing EHR providers, as the integration of new types of data needs to be commercially viable for both implementing organisations and system vendors. Here, genomic information must be stored and coded in a way that allows reclassification over time and associated flagging, for example, in cases where the clinical significance of a variant changes.^22^

In addition, a wider system infrastructure is likely needed to extract and analyse new information. Here, the absence of standardised formats and lack of interoperability of existing systems pose a significant challenge.^23^ Establishing standardised data structures will help maximise benefits through secondary uses of data at scale by enabling insights to be shared across multiple institutions.^23^

While EHRs are fundamental for the advancement of PM platforms, they have inherent limitations.^18^ For instance, in many implementations, EHRs may not clearly distinguish between raw laboratory data and the clinician’s interpretive assessment of those data, particularly when interpretations are entered in unstructured formats or are not systematically recorded.^18^ Given that only a fraction of genomic data are likely to result in clinical action, storing all genomic information within an organisational EHR is likely to be unnecessary. As a result, decisions need to be made surrounding what data should be held where and be accessible to what stakeholders.^18^ Moreover, EHR systems holding PM and genomic data must evolve to align with rapidly emerging evidence. This evolution needs to feed directly into decision support functionality. However, existing decision support systems rely on machine-readable data for generating recommendations, whereas current genomic laboratory outputs are often only readable by humans.^18^

Efforts led by the IGNITE network have shown promise in integrating genomic data into EHRs in individual organisations.^24^ However, the success of these efforts is dependent on the ability to effectively integrate this new information into clinical workflows (see section below).^24^ Prioritisation by organisational leadership and active engagement from clinical users is therefore key.^24^

### System-level transformation of care affecting multiple stakeholder groups

PM platforms are likely to face similar implementation challenges as other complex HIT systems.^25^ But, due to their complexity, they are likely to impact a wider range of contexts, settings and stakeholder groups. For instance, a PM platform may be used in oncology settings to inform treatment decisions, by public health workers to identify populations at risk, by pharmaceutical companies to inform the development of new treatments, by policymakers to inform public health policy and by healthcare organisations to inform resource allocation.^26^ Various health and care settings are also likely to use information held within platforms differently. For example, hospitals are most likely to be interested in developing personalised treatment plans, while primary care is likely to be most interested in preventive activities.^26^ While PM use cases may span different settings, they often rely on shared digital infrastructures, such as genomic databases or interoperable health data platforms. Navigating the competing priorities of different stakeholder groups within such infrastructures may be challenging, as needs may be conflicting, resulting in potential issues surrounding what stakeholder takes priority at any point in time.

An analysis of existing needs and workflows before the deployment of PM platforms is therefore crucial. This should include mapping a wide range of stakeholders early on, considering their diverse interests and positions within the wider health and care system. Transitions of care and care pathways, where potential conflicts may be encountered through data flow across settings, need to receive the most attention as these often present adoption bottlenecks.^25^ Planning how PM platforms align with broader health and care pathways is therefore critical for sustainability.

### Markets, regulation and the innovation ecosystem surrounding PM platforms

Macro-environmental factors including markets and regulatory environments are important when developing and evaluating PM platforms. For example, PM is likely to reshape market dynamics with interventions targeting therapies for small segments of the population.^27^ This may lead to the emergence of new stakeholders, such as biotechnology startups, digital health companies and data platform providers. New business models will be needed to ensure alignment between commercial, organisational and clinical goals. In this evolving landscape, intermediary organisations—for example, those supporting data integration or multi-sector partnerships—may play an increasingly important role.^28^

Regulatory frameworks are struggling to keep pace with the rapid development of PM and AI functionality (which is often linked, such as in AI-driven diagnostics or personalised treatment recommendations), and new approaches are evolving internationally.^15^ The effective exploitation of PM platform functionality requires the creation of an innovation ecosystem, including adequate financing; public legislation and regulation; promotion of multi-sectoral collaboration; and health system, economic resources and human capital.^29^ Data protection and privacy are emerging as significant concerns in this context and need to be monitored. Here, PM platforms will require developing new privacy and data-sharing laws that balance the promotion of data sharing for public benefit with safeguarding individual privacy.^30^ Addressing these challenges will require responsible data-sharing practices, but this is likely to be complicated by the range of organisations and stakeholders involved.^23^

Ethical considerations will need to take a central role in evaluations of PM platforms. For example, there are issues surrounding representation and diversity in PM and genomic research, where certain populations are often over-represented while ethnic minorities are under-represented.^31 32^ The implication is that PM approaches derived from such data will be optimised for those who are represented, with the risk of augmenting existing health inequalities. Many have called for greater diversity in PM research to address health disparities.^33^ Strategies may entail inclusive recruitment strategies, widespread dissemination of results and comprehensive training and education initiatives.^34^

PM initiatives will also require new forms of cross-disciplinary collaboration, and such emerging networks need to be monitored and used to drive the development of communities of practice.^35^ A closer partnership between academia and health system delivery will be essential, signifying a shift towards a more integrated approach where research actively informs and contributes to direct patient care delivery, creating a truly learning health system.

### Integration of PM platforms with organisational processes and user workflows

When evaluating PM platforms, there is a need to assess organisational drivers and processes as well as the integration of systems with user workflows.^36^ Organisational barriers to implementation may, for instance, include the cost of collecting genomic information, which may in turn impact on procurement of systems.^31^ There is also a need to train and educate the workforce and the public in relation to PM.^37 38^ Some work has shown that a key factor contributing to the limited implementation of PM in practice was a lack of training of staff.^39^ In line with this, clinicians often call for more training and education to improve genomic literacy (eg, how to interpret the results of genomic tests).^40^ The public also needs to be more actively engaged than has been the case to date, as currently most PM and genomic medicine efforts are driven by professionals.^32 41^

Monitoring and tracking of unanticipated consequences is critical, as there may be adverse impacts on the provision of care and safety risks associated with implementation.^42^ A key risk in PM and genomic medicine is that these new approaches may inadvertently widen health disparities.^43^ Thus, attention to health equity, stakeholder involvement and diverse needs should be a priority in evaluations of PM platforms. Here, exploring processes before, during and after deployment in various settings can help understand existing practices and assess changes.^44^ Using formative feedback strategies and raising risks early can help address emerging issues as they arise.^9^

It is important to prioritise the needs of end users throughout the development and implementation process of PM platforms. PM platforms require the creation of new workflows,^2^ but there is as yet limited understanding of how data can be effectively integrated with clinical decision-making processes.^45^ Early sharing of prototypes with users can facilitate the identification of potential barriers, and thereby help promote scalability and sustainability of platforms. Incorporating genetic testing aspects into clinical practice also requires a holistic approach that extends beyond individual patients to explore consequences for their relatives.^2^ Additionally, it is important to explore how existing data are integrated with new PM and genomic information and how clinical pathways change with the introduction of new types of data held in platforms.^46^

## Conclusions

PM and genomic medicine initiatives are increasingly integrated into complex platforms aggregating a variety of information that can be used to drive clinical action, organisational decision-making, approaches to public health and policy making. Such complex systems, however, present new evaluation challenges as they hold a variety of novel data sources that need to be translated into action at various levels.

Evaluations need to move away from the currently narrow quantitative focus on individual organisational settings to draw on existing theoretical work and insights surrounding HIT implementation and adoption. Here, particular attention needs to be paid to system-level transformations, including integration with HIT infrastructures; integration across care pathways; and evolving markets, regulation and innovation ecosystems.

Developing evidence-based approaches to evaluation is essential for the safe and effective implementation of PM platforms, so this work is likely to be of interest to those developing and evaluating emerging approaches.
